# Microstructural Study of 17-4PH Stainless Steel after Plasma-Transferred Arc Welding

**DOI:** 10.3390/ma8020424

**Published:** 2015-01-29

**Authors:** Dewei Deng, Rui Chen, Qi Sun, Xiaona Li

**Affiliations:** 1 School of Material Science and Engineering, Dalian University of Technology, Dalian 116024, China; E-Mails: 1320418517chen@163.com (R.C.); sunqi43793870@163.com (Q.S.); lixiaona@dlut.edu.cn (X.L.); 2 Shenyang Blower Works Group Corporation, Shenyang 110869, China

**Keywords:** heat affected zone, 17-4PH, Co-based alloy, microstructure, base metal

## Abstract

The improvement of the surface qualities and surface hardening of precipitation hardened martensitic stainless steel 17-4PH was achieved by the plasma-transferred arc welding (PTAW) process deposited with Co-based alloy. The microstructure of the heat affected zone (HAZ) and base metal were characterized by optical microscope (OM), scanning electron microscope (SEM) and transmission electron microscope (TEM). The results show that there are obvious microstructural differences between the base metal and HAZ. For example, base material is transformed from lath martensite to austenite due to the heateffect of the welding process. On the other hand, the precipitate in the matrix (bar-like shape Cr_7_C_3_ phase with a width of about one hundred nanometres and a length of hundreds of nanometres) grows to a rectangular appearance with a width of about two hundred nanometres and a length of about one micron. Stacking fault could also be observed in the Cr_7_C_3_ after PTAW. The above means that welding can obviously improve the surface qualities.

## 1. Introduction

More and more attention is being paid to precipitation hardening of stainless steel, because of its properties, such as excellent corrosion resistance, high strength, high fatigue resistance and good weldability. 17-4PH is one of the most well-known precipitation hardened stainless steels, which is widely used in oil, gas and aerospace industries. However, its wide application is restricted by its poor tribological properties, which have necessitated the development of advanced surface engineering technologies to improve the surface qualities, such as surface hardness and wear resistance. Co-base alloys with good wear resistance and excellent corrosion resistance are regarded as candidate materials for surface hardening of 17-4PH stainless steel. The good qualities due to the Co-base alloy welding cannot be separated from the fine structure. A microstructureal gradient is observed including the fusion area (the planar region and the bulky dendrite in a direction perpendicular to the weld interface), the transition zone (the dendrite in a multi-direction way) and the fine grain zone near the surface in the coating [1,2,3,4].

A wide range of mechanical properties of 17-4PH stainless steel can be developed through heat treatment at a variety of temperatures because various types of microstructures can be obtained with different heat treatment processes. The martensite formed during cooling from solution treatment temperature as well as the precipitates formed during aging, contribute to the high strength of 17-4PH stainless steel. In the solution annealed condition, its yield strength is typically 880 MPa which increases up to 1200 MPa on aging at 480 °C. However, aging above 480 °C leads to decrease in strength and increase in toughness. The influence of different aging treatments on the mechanical properties is due to the transformation of the microstructure. A few studies have reported on the microstructure of 17-4PH after different heat treatments using TEM (Transmission electron microscope) observation. The corresponding microstructure is composed basically of lath martensite and with numerous precipitation particles dispersed in the martensite matrix. At the same time, it has been claimed that layers of retained austenite could exist along the boundaries of adjacent laths after aging at high temperature. So the heat input during the welding process can have a great effect on the base metal. Study on 17-4PH deposited with Co-based alloy has been reported, but research reports on the effect of the welding process on17-4PH base metal are rarely found. In this paper, the analysis and correlation of the influence of the welding process on the microstructure of 17-4PH is presented [5,6,7,8].

## 2. Experimental Section

In this study, shaft sleeves fabricated from 17-4PH were used as the base material for surface hardening. The chemical composition is shown in Table 1, which is in agreement with the American Society of Testing Materials A705 (grade 630) standard for precipitation hardening forged stainless steel.

**Table 1:** The chemical composition of base metal (%).

Element	C	Si	Mn	P	S	Ni	Cr	Cu	Nb
Content	0.07	≤1.00	≤1.00	≤0.04	≤0.03	3–5	15–17	3–5	0.15–0.45

A surface welding procedure was performed to improve the surface qualities. The plasma-transferred arc welding operation (PTAW) using Co-base alloy stellite12 powder (with chemical composition shown in Table 2) was performed. The PTAW process was used for welding using a current of 160 A, an operating voltage of 24–25 V, a powder feeding rate of 25 g/min, an arc oscillation width of 25 mm, a plasma gas flux rate of 2.5 L/min and a protective gas flux rate of 12 L/min. The shaft sleeve, which was deposited with Co-based alloy, was cut into 15 mm × 15 mm ×15 mm samples. The thickness of the welded surface coating is about 3.5 mm.

**Table 2:** The chemical composition of coating (%).

Element	C	Si	Mn	Cr	W	Fe	Mo	Ni	Co
Content	1.4	1.45	1.00	29.5	8.25	3	1	3	51.4

The microhardness of the base metal and the heat affected zone (HAZ) was measured by a MVC-1000B (Jiming, Shanghai, China) hardness tester with a test load of 500 g and a rhombic indentor; with metallographically prepared samples. The process of specimen preparation for optical microscope and electron microscope is that the sample was polished with Waterproof abrasive paper including 400#, 800#, 1200#, 1500# and then polished using polishing paste, and finally etched. The process of specimen preparation for transmission electron microscopy was as follows: (a) The sample was polished with waterproof abrasive paper including 400#, 800#, 1200#, 1500#, 2000#; (b) The Ion thinning was carried out on the sample. The microstructures of base metal and HAZ were described by a Nikon-MA100 (Nikon, Tokyo, Japan) optical microscope, a ZEISS EVO 18 scanning electron microscope (SEM) (ZEISS, Jena, Germany) and a Philips Technai G2 transmission electron microscope (TEM) (Philips, Amsterdam, The Netherlands); the distribution of the main elements was analyzed by an electron probe microanalysis (EPMA-1600 electronic probe) (Shimadzu, Kyoto, Japan).

## 3. Results and Discussion

### 3.1. Microhardness, Element Diffusion and Microstructure Analysis

**Figure 1:**
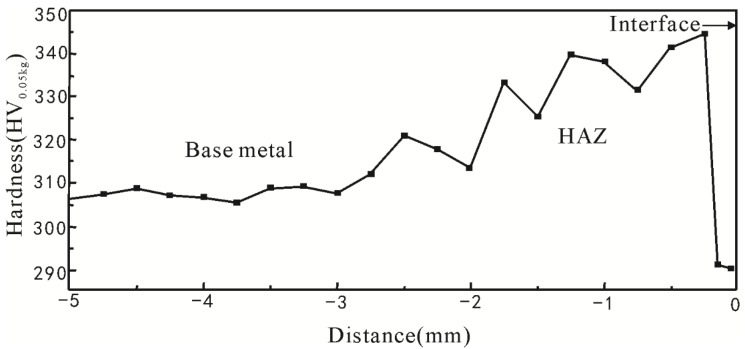
Variations of microhardness from base metal to heat affected zone (HAZ). (Hardness tester with a test load of 0.5 Kg).

Figure 1 presents the Vickers hardness profile of the cross section from the base metal to HAZ in which the interface between the weld region and the heat affected zone is set as zero. As can be seen, the hardness of the base metal, which was not affected by the welding heating cycle, was almost constant and the average value was 307.5 HV. There was an obvious gradient of microhardness in the HAZ. The hardness of HAZ near the interface was at a minimum due to the coarse overheated structures. However, as the distance increased from the interface, solution strengthening (Nb and Cu serve for the solution strengthening) occurred with the formation of quenching martensite, so the hardness adjacent to the interface is at a maximum. However, as the distance increased further, the hardness decreased because of the lower effect of the solution strengthening associated with the reduced number of Cu and Nb precipitates.

**Figure 2:**
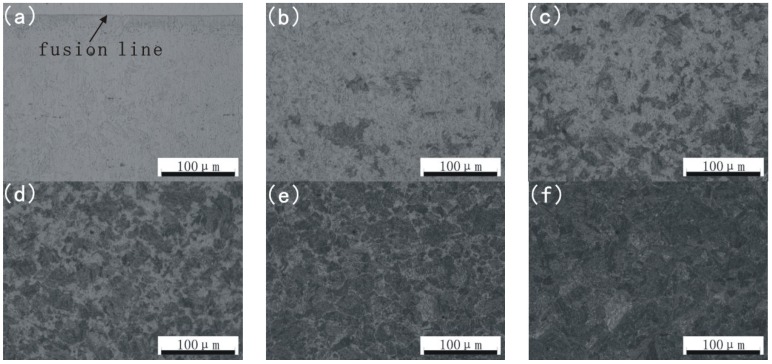
Optical microscope (OM) image of base metal and HAZ distance from fusion line (**a**) 0–0.2 mm; (**b**) 0.75–1 mm; (**c**) 1.25–1.5 mm; (**d**) 1.75–2 mm; (**e**) 2.25–2.5 mm; (**f**) base metal.

**Figure 3:**
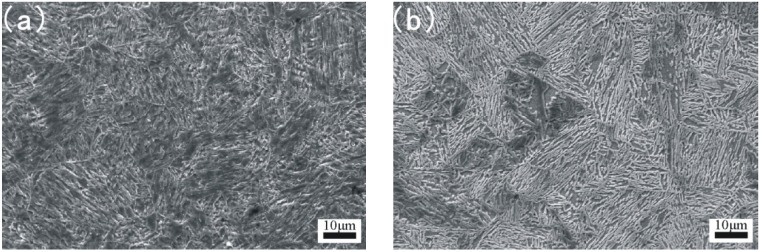
Scanning electron microscope (SEM) image of HAZ and base metal: (**a**) HAZ; (**b**) base metal.

Figure 2 presents the microstructure of the base metal and HAZ observed by OM. The microstructures of 17-4PH from fusion line toward the base metal are shown in Figure 2a–f. The microstructure of the area whose distance from the fusion line is about 0–0.2 mm (Figure 2a) is basically composed of austenite. However, with the distance from the fusion line increasing (Figure 2b–e), the basical microstructure changes gradually from austenite to martensite. Yet, in the base metal, the corresponding microstructure is basically composed of lath martensite (Figure 2f). Figure 2 shows obvious microstructural difference between base metal and HAZ. To demonstrate it further, scanning electron microscope micrographs with a higher magnification are shown in Figure 3. It is noticeable that the microstructure of HAZ (Figure 3a) and base metal (Figure 3b) show a great difference. Martensite lath is dissolved and the grain boundaries of prior austenite become obvious (Figure 3a), which correlates with Figure 2 [9,10]. The microstructure of 17-4PH after PTAW may be affected by two processes, namely, (1) element diffusion during welding and (2) welding heat input and welding cold cycle after PTAW.

**Figure 4:**
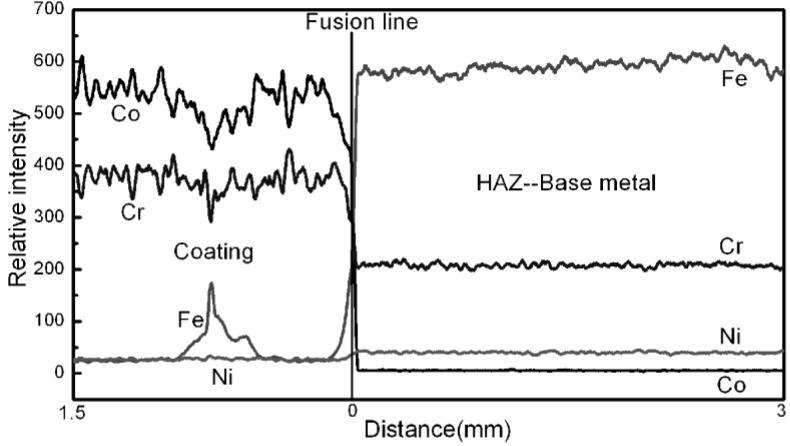
The observation of major element (Co, Fe, Cr, Ni) diffusion from coating to base metal by electron probe microanalysis (EPMA).

EPMA line scanning was carried out to investigate the effect of element diffusion during welding on the microstructure. Figure 4 shows that the content of the major element (Fe, Co, Ni and Cr) on both sides of the fusion line is very different, and the major element (Fe, Co, Ni and Cr) almost does not diffuse between coating and base metal during welding. So the microstructural gradient at HAZ is due to the welding heat input and the welding cold cycle after PTAW, rather than to the effect of element diffusion. This can be explained by the fact that 17-4PH is sensitive to heat treatment and can develop a wide range of properties through heat treatment at a variety of temperatures. Therefore, the heat input and cold cycle during the welding process can have a great effect on the base metal [11,12].

### 3.2. Microstructure Analysis by TEM

OM and SEM are not usually appropriate to reveal details of the microstructure of the martensite matrix. Therefore attention was focused on TEM, which is more effective in determining the microstructure of 17-4PH stainless steel.

#### 3.2.1. TEM Observation of the Base Metal

**Figure 5:**
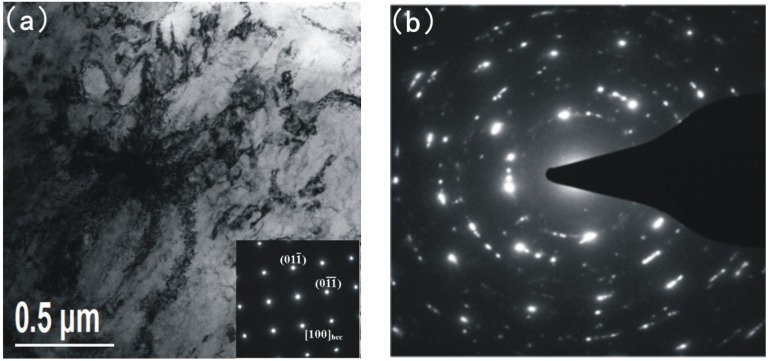
Transmission electron microscopy (TEM) and corresponding diffraction patterns of base metal; (**a**) lath martensite matrix and precipitates; (**b**) corresponding diffraction patterns of (**a**).

**Figure 6:**
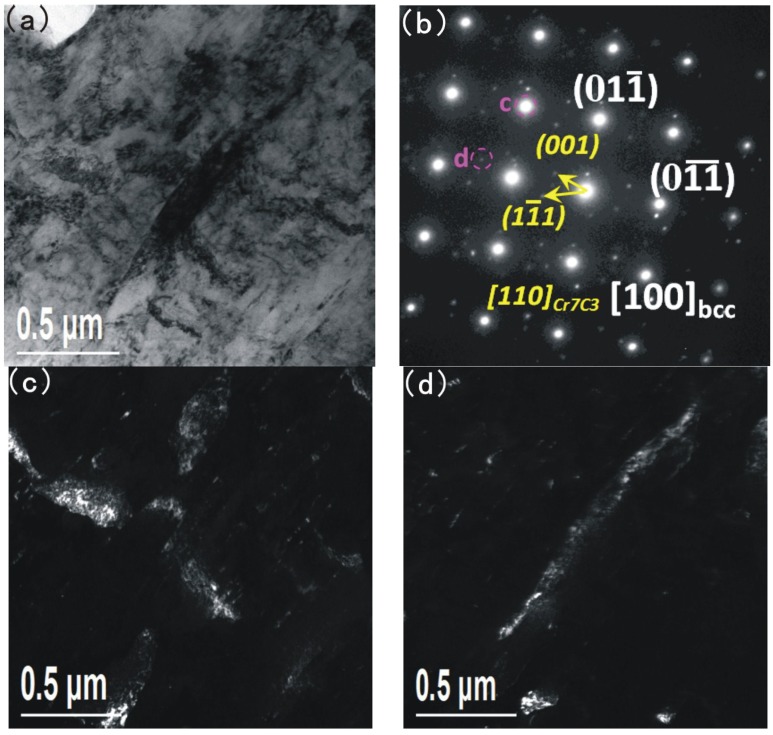
TEM and corresponding diffraction patterns of Cr_7_C_3_ in the base metal; (**a**) bright field image; (**b**) corresponding diffraction patterns; (**c**) and (**d**) dark field images.

**Figure 7:**
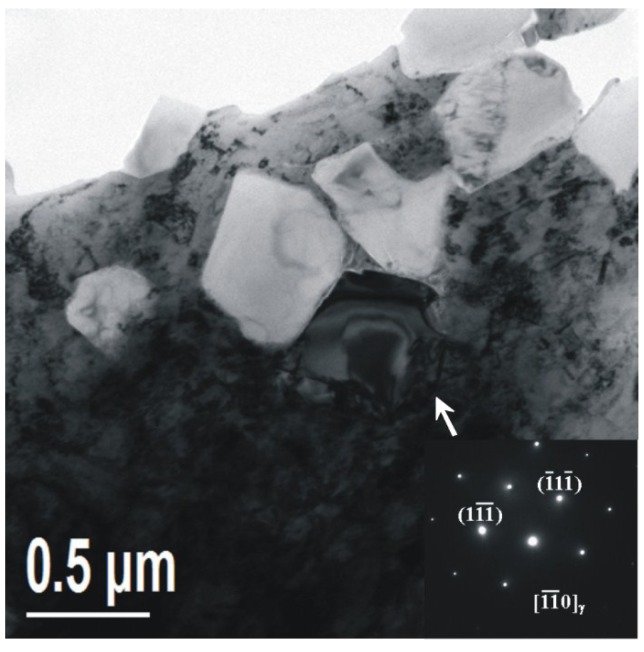
TEM and corresponding diffraction patterns of the austenite in the base metal.

The TEM photographs of the base metal are shown in Figure 5, Figure 6 and Figure 7.

Figure 5 shows the lath martensite matrix, the precipitates dispersed in the martensite matrix and the corresponding diffraction patterns. As can be seen in Figure 5a, the corresponding microstructure is basically composed of lath martensite [13]. Lots of nanoscale precipitation particles are dispersed in the martensite matrix. According to the selected area diffraction pattern (SADP) shown in Figure 5b, the corresponding value of *d* is measured. The results from *d*_1_ to *d*_10_ are 2.061 Å, 1.744 Å, 1.474 Å, 1.357 Å, 1.201 Å, 1.053 Å, 0.945 Å, 0.882 Å, 0.865 Å and 0.801 Å. The measured value of *d* corresponds to PDF-*d* (Å) of Fe (bcc) (2.027, 1.433, 1.170, 1.013, 0.906, 0.827), Cr_7_C_3_ (2.048, 1.754, 1.440, 1.352, 1.210) and Cr_23_C_6_ (2.051, 1.492, 1.256, 1.230, 1.087, 0.931, 0.900, 0.842, 0.815). The matrix is martensite which is Fe (bcc) and the precipitates are identified as Cr_7_C_3_ and Cr_23_C_6_.

A bigger precipitate is found in the martensite matrix in Figure 6. Figure 6a shows the bright field image and Figure 6b shows the corresponding diffraction patterns. The dark field images of the Cr_7_C_3_ and diffraction patterns of martensite are shown in Figure 6c,d respectively. The precipitate is of a bar-like shape, with a width of about one hundred nanometres and a length of hundreds of nanometres. According to the selected area diffraction pattern (Figure 6b), the precipitate is identified as Cr_7_C_3_. Based on the results presented above, it is believed that, Cr_7_C_3_ exists in two forms (the nanoscale precipitation particles dispersed in the martensite matrix and the bar-like shape precipitate).

Austenite is also found in the specimen. As can be seen in Figure 7, there are lots of white blocks in the martensite matrix, with aside length of hundreds on the nanoscale. According to the SADP as shown in Figure 7, the white blocks are identified as austenite. The austenite is like a layer of film in the martensite matrix.

#### 3.2.2. TEM Observation of the HAZ

**Figure 8:**
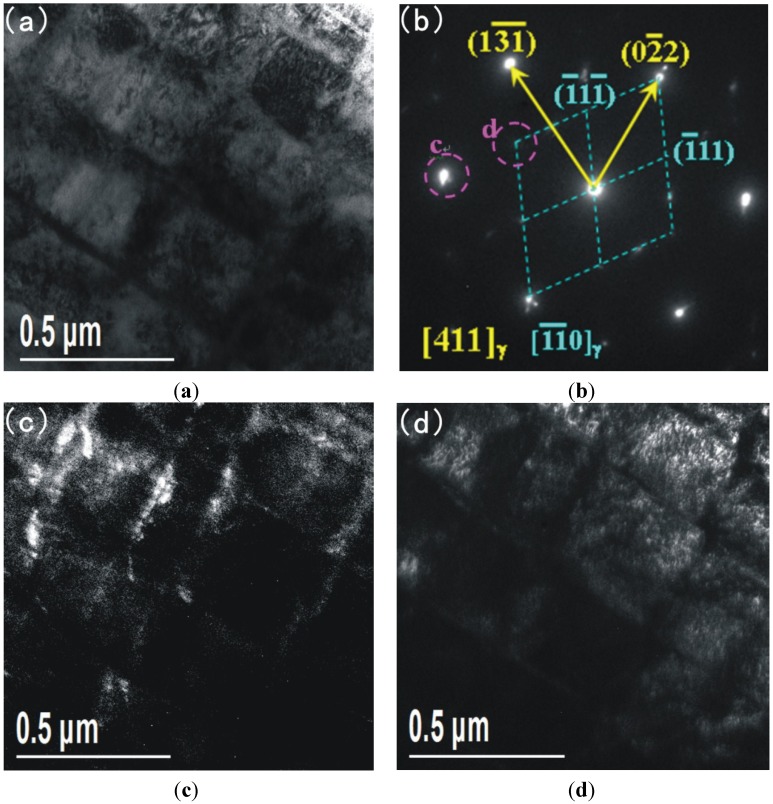
TEM and corresponding diffraction patterns of HAZ; (**a**) bright field image; (**b**) corresponding diffraction patterns; (**c**) and (**d**) dark field images.

**Figure 9:**
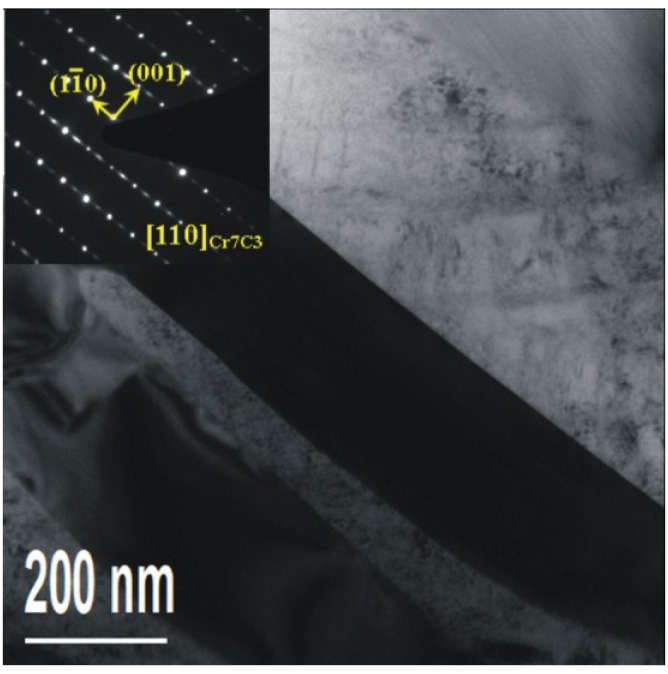
TEM and corresponding diffraction patterns of the Cr_7_C_3_ in the HAZ.

The TEM photographs of the HAZ are shown in Figure 8 and Figure 9.

Figure 8a shows the bright field image and Figure 8b shows the corresponding diffraction patterns. The dark field images of the diffraction patterns are shown in Figure 8c,d respectively. The corresponding microstructure is composed of austenite. The formation of austenite is a result of the welding heat input and welding cold cycle after PTAW. Evident from the analysis presented above, organization transformation from lath martensite to austenite is due to the heat effect during the welding process. Due to the base metal being adjacent to the fusion zone (HAZ) it is effectively annealed by the welding heating and cooling cycles, resulting in the presence of austenite. This is why preheating and post-weld heat treatment (PWHT) are not required to prevent cracking or to restore ductility.

As seen in Figure 9, precipitates are found in the austenite. According to the selected area diffraction pattern (SADP) as shown in Figure 9, the precipitates are also identified as Cr_7_C_3_ (the same structure as the precipitates in the base metal). However, after this welding process, the Cr_7_C_3_ forms a rectangular appearance with a width of about two hundred on the nanoscale and a length of micron size (Figure 9), which is obviously larger than that corresponding to Cr_7_C_3_ in the base metal (Figure 6a). According to the above, the Cr_7_C_3_expands after welding due to the heat affect. In addition, a stacking fault could be observed in the Cr_7_C_3_. The TEM results correspond with the OM results and SEM results.

#### 3.2.3. TEM General Discussion

From the above results it can be inferred that that microstructures between base metal and HAZ have obvious differences. First of all, after the welding process, the lath martensite is transformed to austenite because of the heat effect. In addition, the Cr_7_C_3_ phase changes from a bar-like shape to a rectangular appearance of a bigger size and with the appearance of a stacking fault. The larger size and the changing shape of the Cr_7_C_3_ in the HAZ, and the stacking observed in the Cr_7_C_3_ are due to the welding heat input and welding cold cycle after PTAW.

## 4. Conclusions

The aim of this work was to study the influence of PTAW on 17-4PH base metal. The conclusions of the present study are as follows:

1. After PTAW, obvious microstructural difference between base metal and HAZ are observable by OM and SEM. Additionally the microstructure of 17-4PH after PTAW can be mainly affected in two ways: The first one is element diffusion during welding and the second one is welding heat input and welding cold cycle after PTAW. To investigate the effect of element diffusion on the microstructure, EPMA line scanning was carried out. The result shows that the major elements (Fe, Co, Ni and Cr) almost do not diffuse between coating and base metal, meaning there is almost no effect of element diffusion during welding. Above all, the conclusion can be drawn that the obvious microstructural difference between base metal and HAZ is due to welding heat input and welding cold cycle after PTAW.
2. In order to reveal details of the microstructure of the martensite matrix, attention was focused on TEM. The microstructure of base metal 17-4PH mainly contains martensite, and the existence of precipitates (Cr_7_C_3_ and Cr_23_C_6_) and austenite in martensite is also observed. After PTAW however, the microstructure of HAZ exhibits austenite phase organization transformation from lath martensite to austenite, which occurs because of the heat effect during welding. The existence of Cr_7_C_3_ is still observed after PTAW, but the Cr_7_C_3_ amount is obviously larger than that corresponding to Cr_7_C_3_ in the base metal and a stacking fault could be also observed in the Cr_7_C_3_.
